# Bacteriophages and Diffusion of β-lactamase Genes

**DOI:** 10.3201/eid1006.030472

**Published:** 2004-06

**Authors:** Maite Muniesa, Aurora García, Elisenda Miró, Beatriz Mirelis, Guillem Prats, Juan Jofre, Ferran Navarro

**Affiliations:** * University of Barcelona, Barcelona, Spain;; † Hospital de la Santa Creu i Sant Pau, Barcelona, Spain;; ‡ Autonomous University of Barcelona, Barcelona, Spain

**Keywords:** Bacteriophages, β-Lactamase Genes

## Abstract

We evaluated the presence of various β-lactamase genes within the bacteriophages in sewage. Results showed the occurrence of phage particles carrying sequences of *bla*_OXA-2_, *bla*_PSE-1_ or *bla*_PSE-4_ and *bla*_PSE_-type genes. Phages may contribute to the spread of some β-lactamase genes.

Bacteriophages provide one of the most efficient vehicles for moving DNA sequences between bacterial cells. One consequence of transduction is disseminating sequences that allow bacteria to become more pathogenic and antimicrobial drug resistant (*1**–**5*). In vitro, phages can transduce resistance to imipenem, aztreonam, and ceftazidime in *Pseudomonas aeruginosa* (*4*), methicillin in *Staphylococcus epidermidis* (*5*), tetracycline in *S. aureus* (*3*) and *Actinobacillus actinomycetemcomitans* (*2*). They can also transduce resistance genes from *Salmonella enterica* serovar Typhimurium DT104 (*1*).

Increasing levels of resistance to antimicrobial agents in bacteria, particularly in gram-negative rods resistant to β-lactam antimicrobial drugs, have become evident (*6**,**7*). The major mechanism of resistance that causes clinically important infection in gram-negative bacteria is the production of β-lactamases, which includes chromosome- and plasmid-encoded enzymes (*6**,**7*). Introducing cephamycins and broad-spectrum cephalosporins, such as cefotaxime, ceftazidime, and cefepime, monobactams and carbapenems (*7*) initially stopped the widespread occurrence of classic plasmid-mediated TEM-1, TEM-2, SHV-1, and OXA-1 β-lactamases. Gram-negative bacteria quickly acquired resistance to these drugs by acquiring plasmid-encoded extended-spectrum β-lactamases (ESBLs), cephamycinases, or carbapenemases, among other mechanisms (*7*).

Methods for host-independent detection of transducing phage particles have recently been described. These include phages carrying genes linked to specialized transduction (*8*) and genes likely linked to generalized transduction (*9*). We evaluated genes that encode resistance to β-lactam agents within phage particles present in sewage samples.

## The Study

The study was performed with sewage samples collected during a 6-month period (November 2001 to April 2002). One liter of raw sewage samples was collected monthly from the influent raw urban sewage at three different wastewater treatment plants (Table). Plants 1, 2, and 3 serve populations of 50,000, 400,000, and 1,400,000, respectively. Samples with contamination of animal origin were added to the study to increase information on the presence of phages carrying β-lactamase genes in the environment. Samples were collected from three different abattoirs for poultry, pigs, and cattle.

**Table:** Levels of fecal indicators; fecal coliforms and somatic coliphages in the samples used in this study^a^

Sample^a^	Fecal coliforms (CFU/mL^–1^)^b^	Somatic coliphages (PFU/mL^–1^)^b^	Somatic coliphages in the purified fraction (PFU mL^–1^)^c^	*bla*_OXA_ PCR	*bla*_PSE_ PCR	Other *bla* genes^d^
Plant 1A (10 mL)	9.7 x 10^4^ (2.2 x 10^4^)	5.1 x 10^4^ (3.6 x 10^3^)	2.0 x 10^6^	+^e^	+	–
Plant 1A (10 mL + CsCl)	9.7 x 10^4^ (2.2 x 10^4^)	5.1 x 10^4^ (3.6 x 10^3^)	7.5 x 10^4^	+	+	–
Plant 1A (100 mL)	9.7 x 10^4^ (2.2 x 10^4^)	5.1 x 10^4^ (3.6 x 10^3^)	1.0 x 10^7^	+	+	–
Plant 2A (10 mL)	9.2 x 10^4^ (7.2 x 10^3^)	5.1 x 10^4^ (4.4 x 10^3^)	1.3 x 10^6^	+	+^e^	–
Plant 2A (10 mL + CsCl)	9.2 x 10^4^ (7.2 x 10^3^)	5.1 x 10^4^ (4.4 x 10^3^)	8.5 x 10^4^	+	+	–
Plant 2A (10 mL)	9.2 x 10^4^ (7.2 x 10^3^)	5.1 x 10^4^ (4.4 x 10^3^)	1.3 x 10^6^	+	+	–
Plant 2B (10 mL)	2.0 x 10^4^ (9.6 x 10^3^)	8.3 x 10^4^ (4.6 x 10^3^)	2.5 x 10^6^	+	+	–
Plant 2C (10 mL)	9.0 x 10^4^ (5.0 x 10^3^)	6.2 x 10^4^ (1.7 x 10^3^)	1.5 x 10^6^	+	+	–
Plant 3A (10 mL)	2.5 x 10^5^ (6.0 x 10^4^)	8.9 x 10^4^ (3.0 x 10^3^)	3.0 x 10^6^	+	+	–
Plant 3B (10 mL)	3.0 x 10^5^ (1.7 x 10^4^)	9.3 x 10^4^ (2.0 x 10^3^)	2.5 x 10^6^	+	+^e^	–
Poultry (10 mL)	1.8 x 10^6^ (7.2 x 10^5^)	7.9 x 10^4^ (1.5 x 10^3^)	1.0 x 10^6^	+	+	–
Pigs (10 mL)	3.6 x 10^5^ (1.3 x 10^5^)	1.8 x 10^5^ (1.7 x 10^4^)	6.1 x 10^6^	+	+^e^	–
Cattle (10 mL)	2.3 x 10^4^ (7.2 x 10^3^)	8.5 x 10^2^ (2.7 x 10^2^)	3.5 x 10^4^	+	+	–

^a^Results of nested polymerase chain reaction (PCR) amplification of the β-lactamases *bla*_OXA_ and *bla*_PSE._ Parentheses indicate the protocol used (phage DNA obtained from 10 mL, 100 mL before purification or from 10 mL after CsCl purification). ^b^Arithmetic mean of three independent replicas. Standard deviation in parentheses. ^c^Bacteriophage enumeration of 10 mL of the purified fraction of the sample used for DNA extraction and PCR. ^d^The studied *bla* genes were: *bla*_TEM_, *bla*_SHV_, *bla*_CMY_, *bla*_LAT_, *bla*_MOX_, *bla*_FOX_, and *bla*_VIM_ genes family; *bla*_OXA-1_-, *bla*_PSE-1_-, *bla*_CTX-M_-, and *bla*_OXA-2_-related genes. ^e^Sequenced PCR fragments (see Figure).

Samples were evaluated for levels of fecal contamination by using fecal coliforms and somatic coliphages. Fecal coliforms were enumerated by membrane filter procedures. Somatic coliphages are those which infect *Escherichia coli* WG5 through the cell wall and are detected by standardized methods (ISO 10705-2). They comprise a wide range of phages and are always found in sewage. These phages, currently used to indicate viral fecal contamination in environmental samples, were used in this study to indicate the presence of phages in the samples. Somatic coliphages were enumerated according to the standard method. Values of fecal indicators in the samples are the arithmetic mean of three independent replicas and are summarized in the Table. In all samples, different *Enterobacteriaceae* and *Pseudomonadaceae* strains resistant to β-lactams were present (data not shown), although they were not quantified since they were detected after enrichment cultures were taken.

For these experiments, two samples were used in a first attempt to establish the best method to be applied in the remaining samples. For this purpose, 1 L of raw sewage was collected from the influent raw urban sewage at two different wastewater treatment plants (samples 1A and 2A, Table). Samples were collected and processed within 6 hours of sampling. To partially purify bacteriophages, two assay approaches were used to optimize the method. For both approaches, 10 mL of sewage was filtered through 0.22-mm polyether sulfone (PES) low-protein-binding membranes (Millex-GP Millipore, Bedford, MA) to exclude bacteria and other particles present in sewage and to recover viruses. Samples were then purified with Ultrafree-4 centrifugal filter units of Biomax-PB polyethersulfone membranes with a molecular weight cutoff of 100,000 kDa (Catalog number UFV4 BHK 25, Millipore), recommended by the manufacturer for protein isolation, purification, and concentration of virus. Suspensions containing virus were concentrated at 3,000 *g* for 30 min. Phages in the column were recovered with 300 µL phosphate-buffered saline (PBS). After purification, samples were supposedly free from other microorganisms other than viruses that could interfere with the results (first approach). Ten microliters was used for bacteriophage enumeration, as described previously for somatic coliphages to verify the presence of phages in the purified fraction (Table).

Results of phage enumeration of the fraction obtained after concentration confirmed the presence of bacteriophages (Table). To avoid other nonviral particles at the final stage, which could interfere with results, a second approach was performed on samples 1A and 2A (Table). The bacteriophages present in the 300-µL sample at this stage were purified by CsCl centrifugation at 60,000 x *g*. The band in which we expected a broad range of bacteriophages (*10*), corresponding to a density of 1.46 ± 0.5 g mL^–1^, was collected and dialyzed to remove the CsCl. A final volume of 300 µL was adjusted with PBS. Ten microliters was used for bacteriophage enumeration, as described above for somatic coliphages, to verify that phages were present in the purified fraction. Results of phage enumeration of the fraction obtained with the CsCl densities confirmed the presence of bacteriophages in the purified fraction (Table). Values in PFU mL^–1^ in the purified fraction were lower than those observed in samples without CsCl purification because of some loss of phages in different gradient densities and after the dialysis step. Samples obtained with or without CsCl gradient purification were then processed for DNA extraction and amplification as described below. However, no variation was observed in either sensitivity results or the kind of β-lactamase genes detected because of the purification with CsCl densities (Table), and since CsCl purification implied a reduction in the number of phages detected and used for the polymerase chain reaction (PCR) analysis, this step was not applied in the remaining samples.

To evaluate whether we could detect other β-lactamase genes in a lower concentration of a larger volume of sewage, we also tested for bacteriophages from 100 mL of sewage in samples 1A and 2A (Table). Bacteriophages partially purified from sewage were concentrated by ultracentrifugation, and the pellet, resuspended in 300 µL of PBS, was treated for DNA purification and PCR as described below. Again, no differences were observed concerning the kind of β-lactamase genes detected when testing in parallel 10 mL or 100 mL (Table). The protocol of purification from 10 mL of samples was thus applied in the remaining samples.

In all cases, samples were then treated with an extra amount of DNase to a final concentration of 1,000 U/mL of the water sample, and incubated for 1 h at 37°C to inactivate any free DNA. Previous work performed by our research group to isolate phage particles from sewage (*8*), as well as well-known methods for phage isolation from bacterial strains, showed that lower amounts of DNase (10 U/mL of sample) were sufficient to eliminate any traces of chromosomal DNA in the sample. However, since the concentration of β-lactam genes in sewage is not established, in these experiments we increased the DNase concentration 100-fold, and we performed extra controls with chromosomal DNA to exclude any possibility of contamination.

A 0.1-mL aliquot of each sample was used for direct PCR reaction. Samples containing the virus particles were identified as nondecapsidated samples. DNase was heat inactivated at 95°C for 5 min before PCR amplification. Additionally, sewage samples previously sterilized at 121°C for 15 min were used as negative controls.

Bacteriophage DNA was then extracted with the QIAamp DNA Blood Mini Kit according to the manufacturer’s instructions (Qiagen, Inc., Valencia, CA). We used 200 µL of each sample for DNA extraction, and DNA was finally diluted in 100 µL of double-distilled water (decapsidated samples). Excess DNase was removed by following the steps in the washing section of the Qiagen protocol and by heat inactivation as described previously.

We used 5 µL of phage DNA (≈ 5 ng/µL) for subsequent PCR reactions. The detection of *bla*_TEM_ (expected size of the amplified fragment was 951 bp), *bla*_SHV_ (expected size of 1,016 bp), *bla*_CMY_ (461 bp), *bla*_LAT_ (461 bp), *bla*_MOX_ (408 bp), *bla*_FOX_ (407 bp), and *bla*_VIM_ (801 bp) genes family and *bla*_OXA-1_-related (694 bp), *bla*_PSE-1_-related (420 bp), *bla*_CTX-M-9_-related (857 bp), and other *bla*_CTX-M_-related (394 bp), enzymes was accomplished by PCR as previously described (*6**,**11**,**12*). The *bla*_OXA-2_-related (701 bp) gene was amplified by using the primers OXA2/3 (5´-GCC AAA GGC ACG ATA GTT GT-3´) and OXB2/3 (5´-GCG TCC GAG TTG ACT GCC GG-3´), submitted by D. Sirot, in the same conditions as for *bla*_SHV_. The full size of all *bla* genes was 801–1,146 bp. Finally, a DNA fragment of the gene that encodes the 16S rRNA was also amplified (≈909 bp) as previously described (*13*). Nested PCR using the same pair of primers and conditions was done in all cases.

All decapsidated samples, independently of their origin, gave positive amplification (Table) when primers for *bla*_OXA-2_-related and *bla*_PSE-1_-related β-lactamases and 16S rRNA from eubacteria as positive controls were used (*9**,**13*). None of the DNA sequences of the remaining β-lactamases studied were amplified in any of the samples, indicating no presence or a very low concentration of any other β-lactamases. DNA amplification was not observed in any nondecapsidated samples, a finding that shows that no free DNA was present in the sample after DNase treatment. Despite efforts to increase the sensitivity of the method by increasing the sample volume, we were unable to detect *bla*_TEM_, and *bla*_SHV_, the most frequent enzymes among *Enterobacteriaceae*. The sewage samples studied differed in the presence of phage particles carrying DNA fragments coding for various β-lactamases.

Although the calculations of the number of phages carrying the β-lactamases in the samples could not be determined, a rough estimation based on the minimal number of phages necessary to obtain a positive PCR result in the volume tested assumed that 1–10 phages per milliliter carry a β-lactamase gene in these samples.

The positive amplification of 16S rDNA used as a control of bacterial DNA contamination was useful to confirm the lack of bacterial contamination in the nondecapsidated samples, since results were negative. Moreover, these results confirm the validity of the DNase concentration used. However, in the decapsidated samples, we obtained positive amplification of 16S rDNA. This finding could be explained by the presence of bacterial 16S rDNA in phage DNA attributable to generalized transduction, as previously described by other authors (*9*).

The amplified products of *bla*_OXA-2_-related and *bla*_PSE-1_-related genes of several of the samples were sequenced by the dideoxy method by using fluorescent terminators and an automatic laser fluorescent DNA sequencer (Pharmacia Biotech, Uppsala, Sweden) (Table) (*6*). The deduced amino acid sequence was identical to OXA-2, PSE-1 or PSE-4, and *bla*P from *Proteus* plasmid pCS229 (accession no. JS0755) (Figure). The *bla*_OXA_ and *bla*_PSE_ occur predominantly in *Pseudomonas* isolates, although they have also been reported in members of *Enterobacteriaceae* and *Vibrionaceae*. The β-lactamases most frequently associated with *Enterobacteriaceae*, such as TEM and SHV enzymes, were not detected, whereas OXA and PSE, significantly less frequent, were recovered. OXA and PSE enzymes have often been associated with integron structures, a finding that may play some role in these results. Nevertheless, integrons usually occur on broad host-range plasmids, which may be transferred not only among *Enterobacteriaceae* but also to *Pseudomonas* and *Acinetobacter*. These species may have more transducing phages than *E. coli*. While TEM, encoded by *Tn*3, has been found on IncP plasmids, it is more frequently found on plasmids of a more restricted host range, such as IncF.

**Figure:**
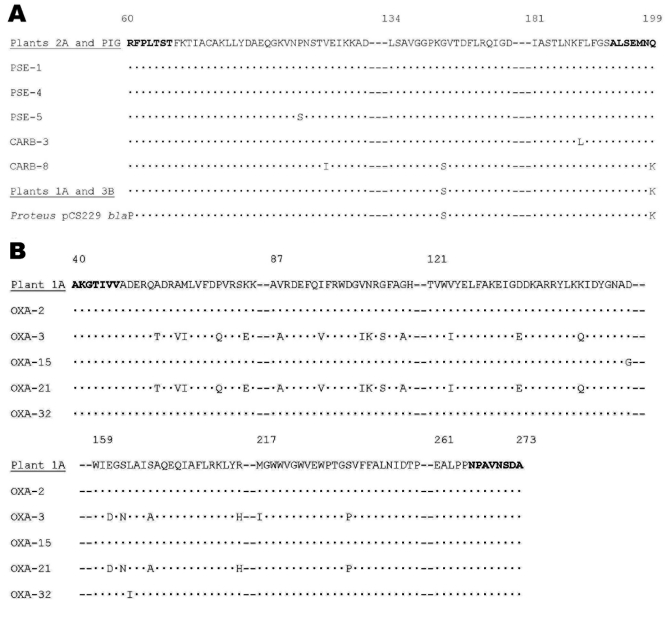
Deduced amino acid sequence of PSE-1 (A), and OXA-2 (B) related enzymes that could be detected by polymerase chain reaction. Amino acids in boldface correspond to the regions of the primers. Dots indicate identical amino acids, and dashes indicate omitted regions with identical amino acids. Numbers correspond to amino acid position. Sequences obtained are underlined. GenBank accession no. of sequences: PSE-1: Q03170, PSE-4: P16897, PSE-5: AAG23870, CARB-3: P37322, CARB-8: AAM92465, *Proteus* pCS229 *bla*P: JS0755, OXA-2: CAA30246, OXA-3: AAC41449, OXA-15: AAB05874, OXA-21: CAA71699, and OXA-32: AAK58418.

## Conclusions

Results reported here show that sewage carries a substantial number of phage particles with various β-lactamase genes. These represent a potential for transduction, and according to the number of particles, the probability of occurrence cannot be overlooked. Our results cannot determine whether generalized or specialized transduction is involved. For this purpose, isolation and characterization of the phage particles would be necessary. Neither can our results show whether the β-lactamase genes detected in phage particles are part of a gene cassette. However, the role of gene cassettes in the emergence and spread of antimicrobial drug resistance has been well-established.

Phages can incorporate genes or groups of genes in their genomes. Their genomic structure is usually modular, with genes of related functions clustered in the genome with their sites of action. This structure allows these genes to be exchanged between related phages by co-infection of host cells and recombination between phage genomes. Some bacteriophages have a broad host spectrum that taxonomically includes very distant species, such as *Sphaerotilus natans*, *E. coli*, and *P. aeruginosa* (*14*). Consequently, infection of these bacteria by phages could be the way the genes, or groups of genes, are able to move over great phylogenetic distances (*15*). A sequence of events in which a phage is infective for two different hosts (A and B) will transfer genes between these hosts. Recombination of these phages in host B with another phage able to infect hosts B and C may facilitate transfer of DNA sequences from host A to host C. Host C could be phylogenetically distant from host A and through this mechanism would acquire the DNA sequences from host A.

Our results indicate β-lactamase genes in naturally occurring phage particles. These genes have been described in several bacteria able to share phages (*7**,**14*) and have also been detected in the sewage samples tested. Therefore, we cannot rule out that phages potentially contribute to the spread of chromosomal genes *bla*_OXA_ and *bla*_PSE_ among *Pseudomonadaceae*, *Enterobacteriaceae*, and *Vibrionaceae*, and the posterior emergence of plasmid-linked antimicrobial-drug resistance cannot be ruled out and is worthy of further investigation. Futures perspectives will be focused on isolating and characterizing single phage particles encoding β-lactamase from genes from sewage and their ability to transduce the character to diverse host strains.
